# Complementary and Alternative Medicines Use during Pregnancy: A Systematic Review of Pregnant Women and Healthcare Professional Views and Experiences

**DOI:** 10.1155/2013/205639

**Published:** 2013-09-30

**Authors:** Abdul Rouf Pallivalappila, Derek Stewart, Ashalatha Shetty, Binita Pande, James S. McLay

**Affiliations:** ^1^Division of Applied Health Sciences, Medical and Dental School, University of Aberdeen, Aberdeen AB25 2ZG, UK; ^2^School of Pharmacy and Life Sciences, Robert Gordon University, Aberdeen AB10 7QB, UK; ^3^Aberdeen Maternity Hospital, Aberdeen AB25 2ZL, UK

## Abstract

*Aims*. To undertake a systematic review of the recent (2008–2013) primary literature, describing views and experiences of CAM use during pregnancy by women and healthcare professionals. *Method*. Medline, Cumulative Index to Nursing and Allied Health Literature, Cochrane Database of Systematic Review Library and Allied, and Complementary Medicine Database were searched. Studies reporting systemic CAM products (homeopathic preparations, herbal medicines, Vitamins and minerals, homeopathy, and special diets) alone or in combination with other nonsystemic CAM modalities (e.g., acupuncture) were included. *Results*. Database searches retrieved 2,549 citations. Removal of duplicates followed by review of titles and abstracts yielded 32 relevant studies. Twenty-two reported the perspectives of women and their CAM use during pregnancy, while 10 focused on healthcare professionals. The majority of studies had significant flaws in study design and reporting, including a lack of appropriate definitions of CAM and associated modalities, absence of detailed checklists provided to participants, the use of convenience sampling, and a general lack of scientific robustness in terms of data validity and reliability. *Conclusion*. To permit generalisability of study findings, there is an urgent need to expand the evidence base assessing CAMs use during pregnancy using appropriately designed studies.

## 1. Introduction

The World Health Organisation defines “complementary and alternative medicine” (CAM) as a “broad set of health care practices that are not part of that country's own tradition and are not integrated into the dominant health care system” [1]. However the scope of CAM is broad and various including therapies such as acupuncture, aromatherapy, herbal, and homeopathic medicines [2]. Whist acknowledging limitations of the published literature (varying definitions of CAM, bias, and confounding), studies undertaken throughout the world have reported women to be large consumers of CAM with prevalence figures of with 56–88% of UK women [3], over 50% of middle aged and older Australian women [4], and over 90% of menopausal Canadian women [5]. Unlike conventional licensed medicines, few CAM approaches to healthcare are supported by robust efficacy, effectiveness, or safety data [6, 7], which raises potential concerns about the use of CAM, particularly in high risk patients such as pregnant women where teratogenicity is a concern [8].

Several limited literature reviews have focused on the use of CAMs by pregnant women [9–11] and recommendations for use by health professionals during pregnancy [9, 12]. While these reviews have concentrated on the findings of relevant CAM-based studies, they have placed less emphasis on critically appraising the core elements of study design and reporting. Limited reviews of CAM recommendations made by healthcare professionals report that CAM approaches, particularly herbal therapy, chiropractic, acupuncture/acupressure, massage, homoeopathy, and aromatherapy are commonly recommended and used in the maternity setting.

We report a systematic review of the primary literature published over the last five years (2008–2012) focusing on the views and experiences of CAM use during pregnancy by women and healthcare professionals, with emphasis on study design and limitations of findings. 

## 2. Method

### 2.1. Search Strategy

A systematic review protocol was prepared as per standard guidelines [13, 14]. The databases Medline, Cumulative Index to Nursing and Allied Health Literature (CINAHL), Cochrane Database of Systematic Review (CDSR) library, and Allied and Complementary Medicine Database (AMED) were searched from January 2008 to December 2012 inclusive, using the search terms (as keywords, title, and abstract) of pregnancy, complementary, alternative, herbal, homeopathic, vitaminmidwife, obstetrician, doctor, physician, woman, patient, views, opinions, experiences, and prevalence. Wildcard symbols, truncation, combinations of search terms using Boolean operators, and alterative spellings were used. Included studies reported either women and/or healthcare professionals' perspectives of prevalence and outcomes of CAM use. Studies reporting systemic CAM products (homeopathic preparations, herbal medicines, vitamins and minerals, homeopathy, and special diets) either alone or in combination with other nonsystemic CAM modalities (e.g., acupuncture) were included. The reference lists from identified papers were scanned for other relevant studies. The search was limited to English language articles. Studies published only as abstracts, letters, or conference proceedings were excluded.

Initial screening of titles was carried out to identify potentially relevant studies, followed by screening of abstracts and then by full paper review. Fifty titles and abstracts were independently evaluated by two reviewers for consistency of inclusion/exclusion. 

### 2.2. Study Review and Data Extraction

Independent quality assessments were conducted by two independent reviewers utilising standard criteria for critical appraisal [15, 16] (Table 1).

Data extracted were; country of study, CAM definitions and scope, sample size and response rate, prevalence of use, perceived effectiveness and safety, and predictors of CAM use. Due to lack of study homogeneity, a narrative synthesis of the results was conducted.

## 3. Results

**Figure 1:**
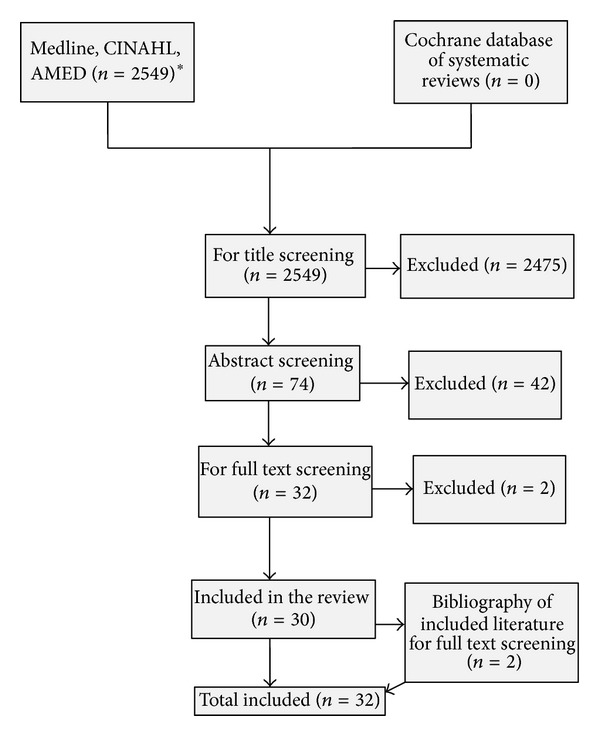
PRISMA flowchart, *after removal of duplicates.

Database searches retrieved 2,549 citations. Removal of duplicates followed by review of titles and abstracts yielded 32 relevant studies. The PRISMA flowchart (Figure 1) illustrates the number of titles, abstracts, and full papers excluded. Twenty-two studies reported the perspectives of women and their CAM use during pregnancy, while 10 focused on healthcare professionals. 

**Figure 2:**
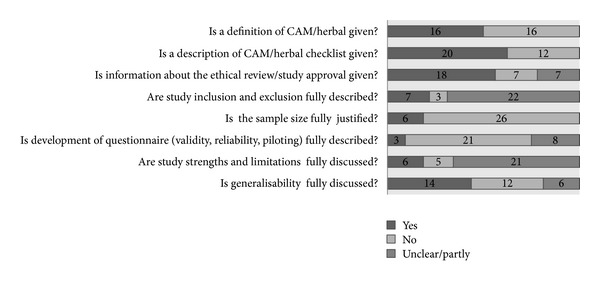
Stacked bar chart representing quality assessment of reviewed studies (*n* = 32).

Included studies were conducted in Europe (*n* = 13), the Americas (*n* = 8), Asia (*n* = 6), Australia and Oceania (*n* = 4), and Africa (*n* = 1). Data were collected by questionnaire (*n* = 21) or structured interview (*n* = 11). Findings of the critical appraisal are reported in Table 2 and Figure 2. Summaries of study details are provided in Table 3 (women) and Table 4 (health professionals). Data should be interpreted with caution due to inconsistency and lack of CAM and CAM related definitions, limitations of study design, and a paucity of outcomes data. 

### 3.1. Studies Assessing Experience and Views of Women (*n* = 22)

Eleven of the 22 studies reported on more than one CAM modality, nine focused on herbals only and two studies reported herbals, and vitamins. 

#### 3.1.1. Definitions and Scope

Of the 11 studies reporting data for more than one CAM modality, only four [20, 28, 31, 38] defined the term “CAM”. Five of these 11 studies [17, 18, 28, 31, 38] provided a detailed and specific checklist of CAM modalities and products to research participants. However, the content and specificity of these checklists were highly variable, precluding direct interstudy comparisons. For example, Adams et al. [17] listed

* “vitamins or minerals, yoga/meditation, herbal medicines, aromatherapy oils, and Chinese medicines” *

while Bishop et al. [18] included

* “treatments, pills, medicines, ointments, homeopathic medicines, herbal medicines, supplements, drinks, and herbal teas”. *

No information on study CAM checklists was provided in the remaining six studies [19–21, 24, 27, 30].

Of the nine studies assessing only herbals use, eight provided a definition of herbal medicine [22, 23, 25, 32, 33, 35–37]. However, no consistent definition was used. For example, Tabatabaee [23] defined “herbal drugs” as

*“all types of products (oral and dermatological) that were manufactured from herbs or contained herbs as the major component” *

while Holst et al. [35] defined “herbal remedies” as

*“any kind of product such as a tablet, a mixture, an ointment or herbal teas which are produced from plants and used to acquire better health”. *

Only five of these nine studies detailed the specific checklist of herbals provided to research participants [22, 26, 35–37]. The content of these checklists varied greatly listing between nine and 40 products, with a free text option to permit participants to add unlisted herbal products. 

#### 3.1.2. Study Design

Only two of the 22 studies assessing the experiences and views of women [18, 19] used the ideal prospective, longitudinal study design, collecting data at several time points during pregnancy and following delivery. However, while Al-Riyami et al. [19] collected data 3 months prior to pregnancy and at each trimester, the study reported by Bishop et al. [18] had a 20-year gap between data collection (1991-1992) and study publication in 2011 raising significant concerns around current validity of data. 

The remaining 20 studies reported collection of retrospective data, thus introducing potential recall bias with implications for data validity and reliability. For example, participants in the study reported by Moussally et al. were required to recall herbal products used during pregnancy up to eight years previously [36]. 

Sample size varied greatly amongst these 22 studies, with participant numbers varying between 139 and 14,541. Only six studies [19, 24, 31, 33, 34, 37] described a sample size calculation detailing estimates of data precision, confidence intervals, and likely response rates; calculated sample sizes varied from 122 to 600. 

The majority of studies used a convenience approach for participant sampling and recruitment, which reduces external validity of the findings. Response rates for questionnaire studies ranged from 39.0 to 99.0%. Two studies attempted to demonstrate homogeneity between respondents and nonrespondents [22, 36]. However, Moussally et al. [36] reported similarity of respondents and nonrespondents in terms of maternal age, gestational age, rate of hospitalisations, pre-natal visits, and comorbidities while Nordeng et al. [22] reported the similarity between respondents and the general Norwegian postnatal population in terms of frequencies of age, parity, and marital status.

#### 3.1.3. Study Findings

Notwithstanding limitations of study design and reporting, it is evident that a significant proportion of women use CAM during pregnancy (see Table 3). Prevalence rates ranged from 5.8% of 4,866 respondents in USA taking herbal or natural treatments [30] to 74.2% of 461 Hispanic women in the USA taking herbals [27]. Only nine of the 22 studies linked CAM usage to pregnancy trimester [18, 19, 23, 25, 26, 29, 31, 34, 37]. Five of these nine studies [18, 19, 26, 29, 37] reported increased use during the later stages of pregnancy, while three studies reported decreased use during pregnancy with the highest use during the first trimester [23, 25, 31]. Forster et al. reported insufficient data to draw any conclusions [34]. 

While 18 of the 22 studies [18, 20–28, 30–32, 34–38] quantified potential predictors of CAM use during pregnancy, only nine used a multivariate approach to analysis [20, 25, 28, 30, 32, 34–37]. Independent predictors for CAM use derived from these nine studies included CAM use prior to pregnancy [20, 28, 35], higher educational attainment [25, 26, 29], chronic disease/meds [28, 32, 36], ethnic background/nationality [21, 28], higher income [21], and age [35]. 

A further major limitation of all 22 studies is the general lack of data on outcomes of perceived effectiveness, safety and overall satisfaction with treatment. While six studies included measures of effectiveness, none provided objective outcome data linked to specific CAM modalities, indications, and trimester of pregnancy. However, in these six studies, respondents' perceptions of effectiveness were generally positive, particularly in comparison with conventional approaches. Mohamed et al. reported that 56.2% of pregnant women thought that CAM was “more efficacious” and while 62.7% of pregnant women in the study by Lapi et al reported “equal effectiveness” [28, 31]. In terms of herbals, Cuzzolin noted that 74.3% of respondents considered these to be “beneficial, reporting good results”, with similar figures reported by Rahman et al. and Fakeye et al. Skouteris et al. commented that almost all (97.3%) users of natural remedies “got completely better” or “got a bit better” [26, 33, 37, 38]. 

Only five studies assessed and reported the potential for possible CAM-related adverse effects (AE's) during pregnancy; however, both intra- and inter-study results were conflicting. While just over half of the respondents in two studies [28, 31] viewed CAM approaches to be safer than traditional medicine, the majority had concerns about the safety of herbals. Kochhar et al. reported that 67.7% of respondents agreed that herbals could harm a baby if taken during pregnancy, and Bercaw et al. reported that a minority of respondents (22%) agreed that herbals were safer to use than prescribed medicines [24, 27]. Similarly, Fakeye et al. reported that 33.4% of respondents believed that herbals possessed no AEs [33]. Although three of these studies did report observed AEs, none provided precise details of the method used for AE identification (specific tick list or open comments). Fakeye et al. reported that 18% of those taking herbals had “some form of unwanted effects” such as vomiting, and dizziness. Cuzzolin and Benoni and Leppee et al. both reported that 3.7% of respondents experienced mild AEs [26, 29, 33]. 

Only one study (Kalder et al.) reported on overall user satisfaction with treatment. The authors limited their report to that “almost all respondents were satisfied” [20]. 

### 3.2. Studies Assessing Experience and Views of Healthcare Professionals (*n* = 10)

Of the ten studies, nine reported data on more than one CAM modality and one on herbals only. 

#### 3.2.1. Definitions and Scope

Of the nine studies reporting data for more than one CAM modality, only four [39, 42, 43, 48] defined “CAM” while eight provided a detailed and specific checklist of CAM modalities and products. Dennehy et al. focused on herbals but neither defined “herbals” nor described a specific checklist [40].

#### 3.2.2. Study Design

Questionnaires were used in all ten studies, with sample sizes varying from 36 (heads of obstetric departments in Croatia) [41] to 1,009 (obstetric gynaecology physicians in USA) [48]. All ten studies used a convenience approach to participant sampling and recruitment with subsequent limitation of external validity of findings. Response rates ranged from 30.2% [40] to 100% [41, 47]; all those with response rates less than 100% did not consider homogeneity of respondents and nonrespondents. 

#### 3.2.3. Study Findings

Six studies [39, 40, 42–44, 47] assessed the views and experiences of midwives while the remaining four studies focused on obstetricians [41, 45, 46, 48]. Despite limitations of study design and reporting, most respondents (other than heads of obstetric departments in Germany) [41] used CAMs in their practice. 

Issues of perceived effectiveness or safety of CAMs were quantified in two studies. Koc et al. reported that 61.2% of midwife respondents in Turkey thought that CAM would be “beneficial”, 24.9% that CAM use decreased pregnancy-related complaints, and 61.2% that CAM use may have AEs [39]. In a study of obstetric physicians in USA, Furlow et al. reported that more than 50% of respondents had positive beliefs on the effectiveness of biofeedback, chiropractic, acupuncture, and meditation whereas 41.2% and 24.9% had positive views on herbals and homeopathic preparations, respectively [48]. Importantly, while the study by Harding and Foureur did not report objective measures of effectiveness or safety of CAM, this study did report that 71.0% of midwife respondents in the USA viewed CAM to be an “essential part of midwifery practice” [43].

## 4. Discussion

Despite a lack of evidence for efficacy and safety, CAM use is reportedly increasing worldwide. Of possible concern is the use of CAM modalities, such as herbals, in high risk patient groups such as pregnant women. The results of this systematic review of the recent published literature highlight the high levels of systemic CAM use, by women and healthcare professionals, during pregnancy.

However, it is clear that the majority of studies assessing CAM use during pregnancy have limitations in terms of both study design and reporting. Specific weaknesses were a lack of appropriate definitions of CAM and associated modalities, the absence of detailed checklists provided to research participants, the frequent use of convenience sampling, and limited detail of considerations of face and content of questionnaire items and test-retest reliability. For these reasons, it was not possible to pool the data from identified studies in an appropriate way to generate meaningful and generalisable conclusions. 

Our systematic review was conducted according to best practice as defined by the Centre for Reviews and Dissemination [13]. Of note each paper was independently reviewed by two authors using standard evidence-based critical appraisal criteria [14]. To ensure that this review was current we are restricted to peer reviewed reports of primary data published during the last five years.

The application of a consistent and useable CAM definition proved to be a major issue. While CAM has been defined by the WHO [1], this definition is vague and open to multiple interpretations, particularly interpretation of “tradition” and “healthcare systems”. Indeed, whether or not a particular therapy is deemed to be CAM may differ between countries, healthcare settings, and specialities. This situation could readily be overcome by the use of standardised, validated checklists. In those studies where checklists were issued to participants, the list of CAM products and modalities included varied widely leading to potentially significant differences in participant product identification. Without a clear and specific definition of each CAM modality and CAM products, it is impossible to perform study comparison or pool data for the purposes of meta-analysis. The lack of definitions and checklists may explain why the reported prevalence of CAM use appears to be highly variable, even within similar populations.

Furthermore, the majority of studies reviewed employed a retrospective method of data collection, with the subsequent limitation of recall bias. The ideal study design which in the case of pregnant women should be prospective and longitudinal, collecting data both before and throughout the course of pregnancy and preferably following delivery should be given consideration in future studies. This would reduce the inherent effects of recall bias and furthermore would permit linkage of CAM use with indication, trimester of pregnancy, and outcome.

Most studies relied on single centre data collection using a convenience approach to sampling. While this may be justified in terms of study logistics, there are clear implications for external validity. Furthermore, a sample size calculation was rarely reported and presenting lacked full statistical justification, thus having implications for confidence in reported outcomes. 

While inferential statistics were employed, there was often a lack of multivariate approaches, which may be one of the reasons for the conflicting results and conclusions in relation to factors influencing the use of CAMs during pregnancy.

There is a need to expand the evidence base assessing CAMs use during pregnancy in terms of prevalence with emphasis on outcomes of effectiveness and potential AEs. Efficacy studies are required for those modalities and products widely used by women during pregnancy and those recommended by healthcare professionals. In addition, qualitative exploration of the reasons for use, factors affecting use, and position of CAMs within the overall treatment hierarchy is warranted. 

## Study Highlights

- Reportedly CAM's are widely used by women during pregnancy.
- What is the quality of recent published evidence reporting the views, experiences of pregnant women and healthcare professionals towards CAM use during pregnancy?
- The quality of the published literature is limited in terms of both study design and reporting.
- There is an urgent need to expand the research based on CAM use in pregnancy. Appropriately designed efficacy studies are required for those CAM modalities and products widely used.

## Figures and Tables

**Table 1:** Criteria for critical appraisal.

(1) Is a definition of CAMs/herbals given?	
(2) Is a description of CAMs/herbals checklist given?	
(3) Is the information about the ethics given (approved/not required)?	
(4) Are study inclusion and exclusion criteria fully described?	
(5) Is the sample size fully justified?	
(6) Is development of questionnaire (validity, reliability, and piloting) fully described?	
(7) Are study strengths and limitations fully discussed?	
(8) Is generalisability fully discussed?	

**Table 2:** Quality assessment of reviewed studies.

Authors, year	Is a definition of CAM/herbal given?	Is a description of CAM/herbal checklist given?	Is information about the ethical/study approval given?	Are study inclusion/exclusion criteria fully described?	Is the sample size fully justified?	Is development of questionnaire (validity, reliability, and piloting) fully described?	Are study strengths and limitations fully discussed?	Is generalisability fully discussed?
Studies of women								
Adams et al., 2011 [17]	N	Y	N	P	N	N	P	N
Bishop et al., 2011 [18]	N	Y	Y	P	N	N	P	N
Al-Riyami et al., 2011 [19]	N	N	U	P	Y	N	P	Y
Kalder et al., 2011 [20]	Y	N	Y	Y	N	N	P	P
Khresheh, 2011 [21]	N	N	Y	P	N	N	N	N
Nordeng et al., 2011 [22]	Y	Y	U	P	N	N	P	N
Tabatabaee, 2011 [23]	Y	N	Y	P	N	N	P	N
Bercaw et al., 2010 [24]	N	N	U	N	Y	N	Y	Y
Broussard et al., 2010 [25]	Y	N	U	P	N	N	P	N
Cuzzolin and Benoni, 2009 [26]	N	Y	Y	P	N	P	P	Y
Kochhar et al., 2010 [27]	N	N	U	N	N	N	P	N
Lapi et al., 2010 [28]	Y	Y	U	P	N	Y	Y	P
Leppee et al., 2010 [29]	N	N	Y	P	N	N	N	N
Louik et al., 2010 [30]	N	N	U	P	N	N	P	N
Mohamed et al., 2010 [31]	Y	Y	N	P	Y	P	P	Y
Chuang et al., 2009 [32]	Y	N	Y	P	N	N	P	Y
Fakeye et al., 2009 [33]	Y	N	Y	Y	Y	N	N	N
Forster et al., 2009 [34]	N	N	Y	Y	Y	P	N	N
Holst et al., 2009 [35]	Y	Y	Y	P	N	N	P	Y
Moussally et al., 2009 [36]	Y	Y	Y	P	N	N	P	Y
Rahman et al., 2009 [37]	Y	Y	Y	P	N	N	P	P
Skouteris et al., 2008 [38]	Y	Y	Y	P	Y	N	Y	Y

Studies of professionals								
Koc et al., 2012 [39]	Y	Y	N	P	N	P	P	Y
Dennehy et al., 2010 [40]	N	N	N	P	N	P	Y	P
Hrgovic et al., 2010 [41]	N	N	Y	N	N	N	N	N
Samuels et al., 2010 [42]	Y	Y	Y	Y	N	Y	P	Y
Harding and Foureur, 2009 [43]	Y	Y	Y	P	N	N	P	Y
Hastings-Tolsm and Terada, 2009 [44]	N	Y	N	P	N	Y	Y	P
Münstedt et al., 2009 [45]	N	Y	Y	Y	N	P	P	P
Münstedt et al., 2009 [46]	N	Y	N	Y	N	P	P	Y
Wiebelitz et al., 2009 [47]	N	Y	N	P	N	P	P	Y
Furlow et al., 2008 [48] USA	Y	Y	Y	Y	N	N	Y	Y

Y: yes; N: no; U: unclear; P: partly.

**Table 3:** Data extraction relating to studies of women (*n* = 22).

Authors, year, country	CAM studied	Stage of pregnancy at point of data collection	Mode of data collection	Response rate (RR, %)	Prevalence in pregnancy
Adams et al., 2011 [17], Australia	CAM	Pregnant	Questionnaire	897 (RR not given)	81% used vitamins and minerals, 16% aromatherapy, and 15% herbal medicines. All significantly higher than nonpregnant women (*P* < 0.001). No data on stage of pregnancy.
Bishop et al., 2011 [18], UK	CAM	Antenatal (weeks 8, 12, 18, 32)	Questionnaire	Total response of 14,115/14,541 (97.1) but varied at each point of data collection	26.7% used a CAM at least once. Most commonly herbal teas (17.7%), homeopathy (14.4%), and herbal medicines (5.8%). Prevalence increased each trimester (6%, 12.4%, 26.5%, no *P* values).
Al-Riyami et al., 2011 [19], Oman	CAM	Antenatal (prenatal, trimesters 1, 2, 3)	Interview	139 but varied at each data collection point	Compared to prenatal, vitamin used increased from 12% to 84–95% across three semesters (*P* < 0.001), herbal medicines from 7% to 16–19% (*P* < 0.05).
Kalder et al., 2011 [20], Germany	CAM	Postnatal	Questionnaire	205/475 (43.2)	50.7% used CAM (including CAM practices). Homeopathy (18.5%), aromatherapy (4.4%). No data on stage of pregnancy.
Khresheh, 2011 [21], Jordan	CAM used in treatment of nausea and vomiting	Currently pregnant or at least one full term pregnancy	Questionnaire	235/290 (81.0)	9.4% used herbal medicines. No data on stage of pregnancy.
Nordeng et al., 2011 [22], Norway	Herbal	Postnatal, within 5 days	Interview	600	39.7% used herbal medicines, 4.3% homeopathy. No data on stage of pregnancy.
Tabatabaee, 2011 [23], Iran	Herbal	Postnatal, 2 days	Interview	513	30.8% used herbal medicines. Tended to be used more in first trimester (no *P* values).
Bercaw et al., 2010 [24], USA	Herbal and vitamins	Postnatal, immediately	Questionnaire	485 Hispanic women (RR not given)	19% used herbal medicines, 47% vitamins. No data on stage of pregnancy.
Broussard et al., 2010 [25], USA	Herbal	Postnatal, 6 weeks–2 years	Interview	4239	9.4% used herbal medicines, the highest for first trimester (6.4% v 5.1 and 5.2%) (no *P* values).
Cuzzolin and Benoni, 2009 [26], Italy	Herbal	Postnatal, within 3 days	Interview	392	27.8% used one or more herbal medicines. Prevalence increased slightly with trimester (no details or *P* values).
Kochhar et al., 2010 [27], USA	Herbal	Not stated	Questionnaire	461 Hispanic women (RR not given)	74.2% used herbal medicines. No data on stage of pregnancy.
Lapi et al., 2010 [28], Italy	CAM	Antenatal, trimester 3	Interview	150	48% used CAM. No data on stage of pregnancy.
Leppee et al., 2010 [29], Crotia, Serbia	Vitamins, minerals and iron	Antenatal	Interview	6,992	56.6% used vitamins and minerals in Croatia, 20.3% in Serbia. Prevalence increased with trimester (no details or *P* values).
Louik et al., 2010 [30], USA	Herbal or natural treatments	Postnatal, within 6 months	Interview	4,866	5.8% used herbal or natural treatments. No data on stage of pregnancy.
Mohamed et al., 2010 [31], Qatar	CAM	Antenatal	Interview	393	67.5% used CAM in first trimester, 37.7% in second, and 28.9% in third (no *P* values). Mainly herbal medicines and food supplements.
Chuang et al., 2009 [32], Taiwan	Chinese herbal	Postnatal	Interview	21,248	33.6% used Chinese herbal medicines. No data on stage of pregnancy.
Fakeye et al., 2009 [33], Nigeria	Herbal	Antenatal	Questionnaire	560/600 (99.0)	67.5% used herbal medicines. No data on stage of pregnancy.
Forster et al., 2009 [34], Australia	Folic acid and other vitamin supplements	Antenatal, ~38 weeks	Questionnaire	588/705 (83.4)	91% took at least one vitamin supplement (mainly folic acid) during pregnancy. Almost all commenced during trimester 1.
Holst et al., 2009 [35], UK	Herbal	Antenatal, more than 20 weeks	Questionnaire	578/1,037 (55.7)	57.8% used herbal medicines. No data on stage of pregnancy.
Moussally et al., 2009 [36], Canada	Herbal	Postnatal, 3–8 years after birth	Questionnaire	3,354/8,505 (39.0)	9% used herbal medicines. No data on stage of pregnancy.
Rahman et al., 2009 [37], Malaysia	Herbal	Postnatal	Interview	210	52.4% used herbal medicines. Majority of use was in trimester 3 (no *P* values).
Skouteris et al., 2008 [38], Australia	CAM	Antenatal, 24–31 weeks	Questionnaire	321 (RR not given)	73.2% used at least one CAM, mostly massage (49.5%), and vitamins/minerals (49.5%). No data on stage of pregnancy.

**Table 4:** Data extraction relating to studies of professionals (*n* = 10).

Authors year, country	Professional group	CAMs studied (authors' terms)	Mode of data collection	Response rate (RR, %)	Prevalence (% recommending for patients)
Koc et al., 2012 [39], Turkey	Midwives	CAM	Questionnaire	129/159 (81.1)	58.9% used CAM in their practice, mostly herbal medicines, diets, and exercises (32.6%, 27.9%, and 28.7%).
Dennehy et al., 2010 [40], USA	Midwives	Herbal medicines	Questionnaire	139/460 (30.2)	66.9% used herbal medicines in their practice.
Hrgovic et al., 2010 [41], Croatia	Heads of obstetrics	CAM	Questionnaire	36/36 (100)	Homeopathy, aromatherapy, massage, moxibustion, phytotherapy, acupressure, reflexology, and Reiki were not used at all (no data on herbal). Acupuncture used at two centres only.
Samuels et al., 2010 [42], Israel	Midwives	CAM	Questionnaire	173/238 (72.7)	70% used CAM in their practice (49.1% massage, 37.0% herbals, and 33.5% homeopathy).
Harding and Foureur, 2009 [43], New Zealand, Canada	Midwives	CAM	Questionnaire	343/648 (52.9)	71.95% recommended or offered CAM often (31%), very frequently (28%) or always (13%). Most common homeopathy, followed by herbal medicines, aromatherapy, and acupuncture (given as frequencies for different patient numbers, e.g., 50% of midwives recommend for 70–100% of their patients).
Hastings-Tolsm and Terada, 2009 [44], USA	Midwives	CAM	Questionnaire	227/500 (45.0)	78% used CAM in their practice mostly herbal medicines (85%), pharmacologic/biologic treatments (82%), and mind-body interventions (80%).
Münstedt et al., 2009 [45], Germany	Heads of obstetrics	CAM	Questionnaire	138/187 (73.4)	100% offered acupuncture, 95.7% homoeopathy, and 50.7% aromatherapy. Decisions to provide CAM were largely made by midwives.
Münstedt et al., 2009 [46], Germany	Head of obstetrics	CAM	Questionnaire	381/946 (40.3)	97.3% offered acupuncture, 93.4% homeopathy, and 76.6% aromatherapy. Decisions to provide CAM were largely made by midwives.
Wiebelitz et al., 2009 [47], Germany	Midwives (lecturers, students)	CAM	Questionnaire	309/309 (100)	63.1% estimated CAM to be “applied frequently” (defined as >25% of their pregnant patients) by midwives. 50–75% estimated homeopathy used “regularly”, 20–40% phytotherapy, and 28-27% hydrotherapy.
Furlow et al., 2008 [48], USA	Obstetric, gynaecology physicians	CAM	Questionnaire	401/1,009 (41.0)	97.6% routinely endorsed, provided or referred patients for at least one CAM modality. 86.4% movement therapies, 80.3% biofeedback etc. 61.4% herbal, and 41.7% homeopathy.
